# Perinephritic Abscess

**Published:** 1883-03

**Authors:** J. W. Holland


PERINEPHRITIC ABSCESS.

This patient, Burk, age thirty-five years, as you see, walks with a limp. The left thigh is slightly flexed on the pelvis, and his body bent forward, inclining toward the left. He entered our ward a few days ago complaining of a dull, deep-seated pain in the left lumbar region. He dates the beginning of his present trouble as far back as last March, when he was seized with pain in the left side and groin, which pain did not leave him for four weeks. He further informs us that he has had repeated attacks like it, which come on suddenly, often with nausea and chilliness, last a few hours or days, and then suddenly disappear.

Mark the locality and the character of the pain. If he were a woman a question of ovarian neuralgia might arise. As it is, we can come to but one conclusion, and that is that he has been subject to nephritic colic.

For some years, at regular periods of three weeks or more, some solid mass has passed down the ureter, causing the sudden griping pain in the left groin, the chilliness and nausea. He has often observed a red color about his urine, though no gravel has been voided. After the colics he has noticed copious discharge of whitish urine. The attack of last March was of much longer duration than any previous one; the colic slowly ceased, and in its place a sore feeling was left. He shortly became aware of a tender swelling in the left side. This is the history of a case of nephritic colic ending in obstruction of the ureter. He says that for years he has had some uneasiness in the small of the back, and, on sudden jarring movement, an acute pain will strike him there. I have examined his urine and find it purulent, There are no bladder symptoms, no discharge in the intervals of micturition, and no tube-casts, hence I infer that the pus comes from the pelvis of the kidney and not from any other portion of the urinary tract. When he is stripped you perceive that the left ilio-costal interval is obliterated. I cannot grasp the soft structures in the left side because of the construction and deformity. There is a slight difference of contour between the two loins, viewed from behind, but no fluctuation, redness, or "pointing" at the site of the tenderness. He informs us that there was a very decided lessening of pain and swelling about six weeks ago. This swelling was probably due to retention of the urine in the pelvis of the left kidney, and to the further formation of an abscess. That it was not simply hydro-nephrosis the following facts show: During last summer he suffered greatly from chills and night-sweats, and lost twenty-five pounds in weight. Under the circumstances these symptoms, hectic and emaciation, unmistakably indicate suppuration.

But you ask, What has become of the pus? Why does not the abscess point? My answer is that it did point, and did discharge itself at the time when the swelling and pain subsided. A nephritic abscess may discharge in many different directions. It may find an outlet through the abdominal parietes into the bowels, into the hip joint, below Poupart's ligament, into the bladder, into the pleura, or into a bronchial tube. About six weeks ago he had a slight cough, without expectoration, which lasted for a few days, and one night he awoke in a violent fit of coughing and expectorated a great quantity of matter, which he says came so fast that it almost choked him. This cough has continued since. It is decidedly worse when he lies on the back or left side. The sputum is purulent and very unpleasant in odor; not exactly urinous, but worse. He says that the cough brought relief to his pain, and made walking easier. You inquire, What relation is there between the pylo-nephritis and his walking? Let me remind you of the anatomical relations of the kidney. Remember that it lies directly in front of the psoas muscle. When the perinephritic cellular tissue is inflamed or receives the contents of the ruptured pelvis of the kidney, every movement of the parts to which the muscle is attached causes pain. In consequence there is a reflex tonic contraction of that muscle, hence the limping, hence the bent thigh.

The obliteration of the ilio-costal interval is partly due to this, and partly also to the like action of the transversalis and the quadratus lumborum. I suppose that the abscess burrowed upward under the arch of the diaphragm and entered the thorax. As there was no pleuritic stitch then, and there is now no indication of pleuritic effusion, it is fair to infer that the pus kept to the mediastinal space. There is no dullness of the lower lobe of the left lung. In fact there are no abnormal pulmonary signs whatever, except moist râles in the larger bronchi. It is quite plain that the final irruption took place into the left bronchus. He is thin, sallow, cachectic, losing flesh day by day, and unless some other vent is found for the pus I fear that his lungs will not remain sound long. Hoping to strike the abscess and empty it by the loins, we introduced an aspirator needle for two and a half inches through the quadratus muscle at the point he indicated as the tenderest. It is not necessary to wait for fluctuation. With the assurance we already have of suppuration, it is advisable to evacuate the abscess through the least injurious channel, namely, the posterior abdominal wall, even if several inches of muscle must be penetrated to effect our object. We failed to reach the abscess in our explorations, which have at least done him no harm. The operation is back of the peritoneal reflection, so that there is no risk of peritonitis. For the present we will be content with the administration of tonics and supportives to neutralize the effect of the chronic discharge. He takes cod-liver oil with hypophosphites and whiskey and nine grains of quinine daily. Warm fomentations are applied to the loins.

Perhaps the future may develop some new indication for surgical treatment by the pointing of the abscess externally.

*Note.* During the month of November, Dr. J. M. Holloway aspirated twice to a depth of three and four inches with negative results. In December the abscess broke into the bowels, yielding a great quantity of the pus. Since then the cough and the lump have disappeared, and the patient has gained flesh and strength week by week.—*J. W. Holland, in Louisville Med. News.*

